# Using Intervention Mapping to Develop Health Education and Health Policy Components to Increase Breast Cancer Screening and Chemotherapy Adherence Among Syrian and Iraqi Refugee Women in Beirut, Lebanon

**DOI:** 10.3389/fpubh.2020.00101

**Published:** 2020-04-15

**Authors:** Lea Sacca, Christine Markham, Johny Fares

**Affiliations:** ^1^The University of Texas Health Science Center at Houston School of Public Health, Department of Health Promotion and Behavioral Sciences, Houston, TX, United States; ^2^The University of Texas MD Anderson Cancer Center, Department of Infectious Disease, Infection Control, and Employee Health, Houston, TX, United States

**Keywords:** intervention mapping, breast cancer, education, policies, refugee women

## Abstract

**Introduction:** Despite the continuous increase in the incidence of metastatic breast cancer among Syrian and Iraqi refugee women residing in camp settings in Lebanon, mammography and chemotherapy adherence rates remain low due to multiple social, economic, and environmental interfering factors. This in turn led to an alarming increase in breast cancer morbidity and mortality rates among the disadvantaged population.

**Methods:** Intervention mapping, a systematic approach which guides researchers and public health experts in the development of comprehensive evidence-based interventions (EBIs) was used to plan a health education and health policy intervention to increase breast cancer screening and chemotherapy adherence among Iraqi and Syrian refugee women aged 30 and older who are residing in refugee camps within the Beirut district of Lebanon.

**Results:** The generation of the logic model during the needs assessment phase was guided by an extensive review of the literature and reports published in peer-reviewed journals or by international/local organizations in the country to determine breast cancer incidence and mortality rates among refugee women of Syrian and Iraqi nationalities. The underlying behavioral and environmental determinants of the disease were identified from qualitative and quantitative studies carried out among the target population and also aided in assessing the sub-behaviors related to the determinants of breast cancer screening and chemotherapy completion as well as factors affecting policy execution to formulate performance objectives. We then developed matrices of change objectives and their respective methods and practical applications for behavior change at the intrapersonal, interpersonal, organizational, and societal levels. Both educational components (brochures, flyers) and technological methods (videos disseminated via Whats app and Facebook) will be adopted to apply the different methods selected (modeling, self-reevaluation, consciousness raising, persuasion, and tailoring). We also described the development of the educational and technological tools, in addition to providing future implementers with methods for pre-testing and pilot-testing of individual and environmental prototype components.

**Conclusion:** The use of intervention mapping in the planning and implementation of holistic health promotion interventions based on information collected from published literature, case reports, and theory can integrate the multiple disciplines of public health to attain the desired behavioral change.

## Introduction

Breast cancer is the most common type of cancer diagnosed in women globally, and the second leading cause of cancer-related death among females (1, 2). This health concern significantly affects vulnerable populations, particularly refugees, who fled their war-torn countries in search of a safer and healthier life (1). The Middle-Eastern region is currently suffering from the highest rates of breast cancer, especially in countries like Lebanon, Jordan, and Turkey, which are reported to be the three main host-communities for Syrian refugees, following the 8-year war in Syria that was fueled by the Arab Spring (1, 3, 4). It is estimated that approximately 72% of cancer deaths occur annually in low- and middle- income countries (1, 3, 5). Around 200,000 breast cancer fatalities were recorded in 2015 among Syrian refugees residing in host communities. The high mortality rate was mainly attributed to the lack of effective, affordable, and accessible preventive (screening, clinical diagnosis) and treatment (radiotherapy, chemotherapy, medications) measures (1). In 2018, Lebanon was ranked first among the Middle East and North Africa (MENA) region for having the largest influx of Syrian refugees in proportion with its overall national population, 183 refugees per 1,000 Lebanese citizens (3). A report released by the Lebanese Ministry of Public Health in 2016 to investigate the impact of refugees from Syria and Iraq on breast cancer incidence rates in Lebanon highlighted an increase of 37.6% in the number of annually reported breast cancer cases. Moreover, in 2015, 41% of patients with breast cancer diagnosed and treated at the American University of Beirut Medical Center (AUBMC) were Iraqi and Syrian refugees, of whom 24% had metastatic cancer (3). These results represent an increase of 2,821 new cases of breast cancer in Lebanon by 2015 compared to 1,758 cases in 2008. Based on 2015 data from the Lebanese Ministry of Public Health and the AUBMC's database registry, of the total 628 cases treated at AUBMC, 372 were Lebanese and 213 were Iraqi refugees. Over one quarter (28%) of the Lebanese female cases were detected through mammography and 15.3% were metastatic cases at diagnosis. However, among Iraqi refugees, only 4% were screen detected, and 24.4% were diagnosed at stage IV breast cancer (6).

A combination of personal determinants such as low self-efficacy, low knowledge, high perceived barriers, and low perceived susceptibility; and environmental factors such as low awareness of physicians regarding the severity of metastatic breast cancer among refugee women, lack of health literate and culturally competent educational skills, and issues related to the local healthcare system (unavailability of funding for chronic disease screening and treatment for refugees, high costs of medical services, lack of properly educated nurses, and lack of transportation means from camps to hospitals) have been negatively affecting screening and chemotherapy completion rates among the target population (1, 3, 7–16). Aside from the lack of funding allocated by the main refugee organization in the country, United Nations High Commissioner for Refugees (UNHCR), the absence of human right policies at the societal level to protect the integrity of refugees and ensure the equitable receival of urgent medical care by disadvantaged populations amidst prevailing political corruption has worsened mammography and treatment rates by contributing to the stigma associated with e refugee status in the host community (17, 18).

The most salient and changeable personal determinants that adversely affect reported screening and chemotherapy adherence estimates among Syrian and Iraqi refugee women in Lebanon encompass the following: low self-efficacy to perform mammography and complete recommended chemotherapy treatment due to social and religious barriers, lack of knowledge about susceptibility to disease, and high perceived barriers including financial and communication issues with physicians in host communities (7, 8, 10, 15, 16).

In 2006, the Breast Health Global Initiative (BHGI) developed and published resource-stratified breast cancer guidelines to address the quality of care received by breast cancer patients in low and mid-resource countries. Several levels are addressed through these guidelines including awareness and education, prevention, early detection and treatment, and overall healthcare systems and public policy with the goal of aiding countries in the better effective utilization of available resources while working on ameliorating other lacking resources, infrastructure, and human capabilities. These guidelines identified four distinct levels of resource availability (basic, limited, enhanced, maximal), each of which have a cumulative set of recommendations that take into account the nature of the country's regulatory environment, the cancer care workforce, and other intervening societal factors (19, 20). Subsequently, in 2016, the National Comprehensive Cancer Network (NCCN) launched a program that incorporates the BHGI resource stratification guidelines and released the NCCN framework for resource stratification which includes updated definitions for each resource level (3). Despite the availability of internationally recognized resource-stratified guidelines, and the continuous advocacy efforts of global health experts to allocate the needed resources based on these guidelines to provide cancer care for patients in low-income and war zone countries, rarely any interventions have been implemented to address this alarming health issue.

Interventions of this kind are specifically recommended in the Middle East, where the influx of refugees and the ongoing financial crisis have debilitated the healthcare system in host communities at a national and regional scale (1, 3, 19). This article describes the application of Intervention Mapping (IM), a systematic theory- and evidenced approach to health promotion planning, to develop a breast cancer screening education and chemotherapy adherence policy program to promote mammography acceptability and treatment completion among Syrian and Iraqi refugee women residing in camps located in the Beirut district of Lebanon. The intervention focuses on the basic and limited levels of the BHGI framework. The effort was carried out in collaboration with Health Promotion professionals from the Lebanese Ministry of Public Health and Lebanese physician experts who are knowledgeable about the overall health situation of refugees in the country.

## Methods

### Intervention Mapping

IM is a systematic approach to develop and implement theory- and evidence-based health promotion interventions to tackle health issues from an ecological perspective (20). The IM protocol defines the six steps of IM starting from problem identification to solution generation and implementation. Each step incorporates several tasks that need to be completed to create a product that guides implementation of the subsequent step. Even though all six steps are interlinked, IM is an iterative rather than a linear approach, providing program planners with the opportunity to move back and forth between tasks and steps to make necessary corrections as new knowledge and perspective are gained throughout the ongoing evaluation process. Yet, the process is still considered cumulative, since inattention to the progress of one step can jeopardize the effectiveness and compromise the validity of the entire intervention (21). Many breast cancer screening programs targeting low-income populations in the United States have successfully used IM to guide the different steps of intervention development and implementation (22–25). In this article, we describe the first four steps of the IM process (needs assessment; performance and change objectives; program design; and program production). The PRECEDE model adapted in IM is a comprehensive structure used to evaluate health needs for the purpose of guiding the design and implementation of effective and focused public health programs. It involves the assessment of several community factors including social determinants, epidemiological determinants, as well as behavioral and environmental determinants, which will be carried out throughout the first four steps of intervention mapping (26). In step 1, the PRECEDE model was applied to conduct a comprehensive needs assessment. This step will be based on a review of previous literature and case reports highlighting the emerging need for cancer prevention and treatment measures among refugees in Lebanon. In step 2, the overall behavioral and environmental goals were highlighted, and matrices were developed to combine the health-promoting behaviors, the environmental policy-related factors, and their respective determinants to generate change objectives. During step 3, theory-based behavioral and environmental change methods were selected based on the change objectives to influence the chosen determinants. Finally, we proposed a plan to pre-test the program in step 4.

### Theoretical Underpinnings

To focus on the major risk behaviors and environmental barriers influencing the adoption of the health-promoting behavior, the Integrated Behavioral Model constructs were used which encompasses constructs from the most frequently used theories (Theory of Reasoned Action, Theory of Planned Behavior, Social Cognitive Theory, and Health Belief Model) such as: perceived severity, perceived susceptibility, self-efficacy, skills, perceived barriers, and outcome expectations (27). By taking into account these constructs, a better understanding of the determinants related to screening and chemotherapy was established, which in turn enabled a careful selection of theory-based methods to achieve behavioral and environmental change (27).

## Results

### IM Step 1: Needs Assessment

Recent literature published in 2017 show that the 72% of cancer deaths occurring in low- and middle-income countries can be attributed to late diagnosis of the chronic disease followed by the availability of affordable low-quality treatment rather than optimal treatment. In 2015, an estimated 200,000 Syrian refugee cancer patients were reported dead due to deficiencies in first-line treatment and the lack of a sufficient number of specialized physicians in both host countries (Lebanon, Jordan, and Turkey) and the countries of origin (Syria and Iraq), which resulted in a loss of therapeutic and survival benefits (1). Recipient countries are not offering refugees residing in camp settings with the basic medical services they are in dire need of as a result of the political unrest and the financial crisis ruling over the region (1, 3, 28). Moreover, UNHCR has no budget guidelines for chronic disease management and treatment as compared to the funds allocated to the control of infectious diseases in unsanitary camp settings (1, 3, 6, 29). Therefore, we focused the intervention on reaching this specific target population (Syrian and Iraqi refugee women residing in Beirut refugee camps in Lebanon) through a collaboration with the main health agents and health institutions in the country who will be part of the planning group.

**Figure 1:**
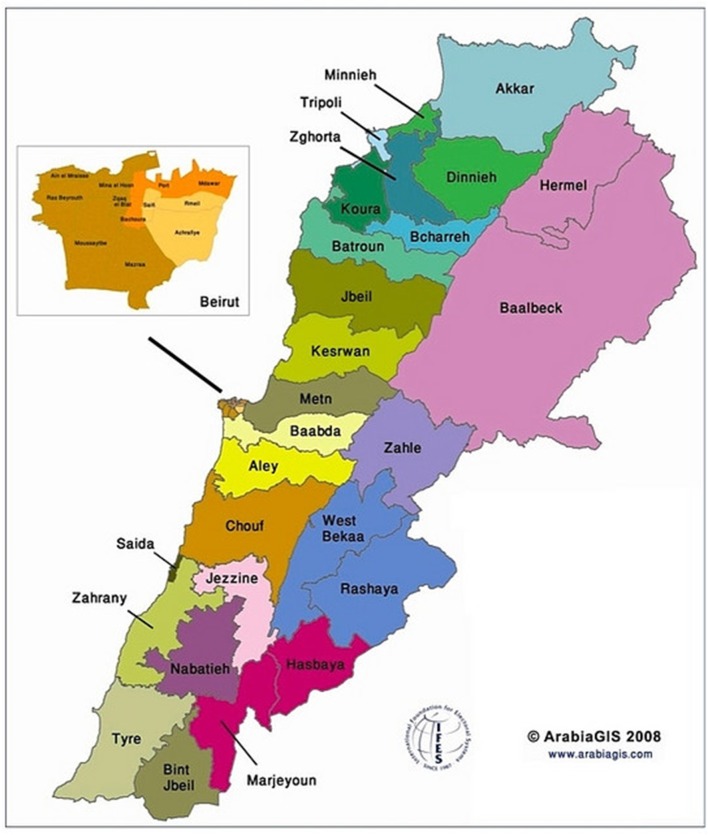
Distribution of districts and regions across Lebanon.

An estimated 338,915 of Syrian refugees in Lebanon reside in refugee camps located in the Beqaa governorate of the country compared to a total of 249,110 Syrian refugees who are living in the Beirut district camps. Around 6,100 Iraqi refugees were reported to be dispersed across these two regions of Lebanon (30–32). The Beirut Governorate encompasses only one district, the city of Beirut; therefore, for the sake of Beirut, the terms “governorate” and “district” can be used interchangeably. As for Beqaa, the governorate incorporates three different districts: Zahle, Rashaya, and West Beqaa, and one of the UNHCR local offices is situated in Zahle. The Zahle district is considered a focal point for data collection purposes (32–34) (Figure 1). In terms of geography, the distance between the Beqaa and Beirut governorates is 39.99 miles (64.36 km), which takes approximately an hour and 5 min to travel from one district to another (35). In 2018, UNHCR disclosed in its response plan for the Lebanese crisis for the time period 2017–2020 that displaced Syrians in Akkar, Baalbek, and Beqaa suffer mostly from economic, social, and health-related barriers, where four out of five individuals fail to satisfy their daily basic needs (32, 36). The Primary Health Care Center (PHC) distribution rate in Beqaa is 6% in contrast to 10% in Beirut, while two public hospitals are available in each of the governorates (37, 38). The reason behind the selection of Beirut governorate as the location for the implementation of the intervention is the greater availability of PHCs and the high allocation of funds to the Rafic Hariri University Hospital, one of the two public hospitals in the area, which renders this medical institution as a main establishment for the receival of medical services among Lebanese citizens and Syrian refugees (39). Moreover, the Beirut governorate includes a single city compared to the three districts in Beqaa, which makes it logistically easier to carry out the intervention in terms of accessing the refugees (localized in one district rather than three) (34). Following the expected success of the intervention, we project a greater dissemination of the project to the remaining refugee camp settings in Lebanon.

Diverse stakeholders will be part of the planning group to ensure a representative sample of the eclectic political, health, social, religious, and economic sectors related to the health problem. Program developers and funders consist of health professionals (oncologist, radiologist, psychologist, behavioral scientists, epidemiologists), translators, interpreters, and community leaders, who will aid in planning and implementing the different tasks leading to an effective health intervention that targets breast cancer while integrating all social, religious, cultural, communication, and behavioral aspects in program design and delivery. Potential sponsors include medical equipment (new available screening technologies) and pharmaceutical companies (chemotherapy drugs); therefore, representatives from such corporations will also be members of the planning group. Lebanon is a highly politicized country, which renders the inclusion and involvement of diverse governmental sectors a necessity for the long-term success of the proposed program. Various governmental departments (Ministry of Public Health, Ministry of Social Affairs, Ministry of Higher Education, & Ministry of Public Works and Transport) are in direct competition for funding measures due to financial and resource constraints that are associated with religious and political issues in the country (40). Hence, collaboration across departments must be secured to ensure effective management of funds to develop and implement a new health promotion intervention that can be maintained over time to satisfy the rising needs of disadvantaged population groups within the Lebanese society (41). Local institutions (American University of Beirut-Global Health Institute and the Lebanese Breast Cancer Foundation) who are known to be active planners and implementers of national health-related campaigns can act as both sponsors and developers. Sponsorships and grants are expected to be provided by the multiple sponsors to aid MOPH and the designated UNHCR departments in policy execution both before and after the policy is officially implemented since the legal and political structure of the intervention is crucial for its long-term sustainability. In case the policy execution takes longer than expected, other local and international institutions will be asked to contribute financially to ensure the long-term sustainability of the intervention. The team of health professionals (physicians, public health workers, and nurses) who will be carrying out the intervention, will collaborate and coordinate efforts with the board of directors of public hospitals and primary healthcare centers since the main program objectives involve the assurance of affordable, accessible, and quality screening measures (mammography) along with subsidized treatment (chemotherapy and medications) which will be available for refugee women in these public healthcare establishments. Finally, refugee women representatives, who have successfully battled breast cancer, are suffering from breast cancer, and who are not impacted by breast cancer, will be involved as part of the planning group since their perspectives and opinions are invaluable to the success of the program.

Since limited empirical data is available on Syrian and Iraqi refugee having breast cancer in the Middle East and North Africa (MENA) region, a thorough and extensive review of case reports, theoretical literature, and firsthand data was carried out to identify the main factors prohibiting the target population from screening and proper chemotherapy treatment. The database search was guided by the previously mentioned constructs of the Integrated Behavioral Model to determine behavioral and environmental determinants for change.

### Personal Determinants and Environmental Factors From Descriptive and Experimental Studies

In 2011, Saadi et al. carried out a qualitative study among a group of Iraqi refugee women who have immigrated to the United States to examine their perspectives on preventive care and their perceived barriers to breast cancer screening. At the personal level, the main emerging themes included: reliance on God to prevent illness; preventing disease as a function of nutrition and cleanliness not doctors; fear of pain during mammography; and fear of receiving a cancer diagnosis. On the other hand, the environmental level included accessibility (testing centers far away), quality (low-quality care in war-zone countries), and availability (limited number of physicians to provide needed care) problems (42). Being part of the Muslim religion and having extremely conservative beliefs were also noted as a barrier since mammography is regarded as inappropriate because prevention efforts are considered anti- fatalism or acting against “God's will” (15, 40). A more recent descriptive cross-sectional study carried out by Al Qadire et al. (7) among Syrian refugee women in Jordan used the Cancer Awareness Measure (CAM) as a tool to look at awareness of cancer symptoms, anticipated delay, and barriers to seeking help. The main reported concerns at the intrapersonal and interpersonal levels were the lack of awareness about cancer symptoms; low knowledge about cancer risk factors; fear of the unknown; worrying about what the doctor might say; being too scared or embarrassed; and difficulty talking to the doctor or wasting his time. Other major environmental factors include the lack of insurance coverage to cover the cost of medical care; safety concerns; and settlement difficulties (7). To address these recurring themes and issues prohibiting effective access to quality breast cancer screening and chemotherapy, some interventions have been carried out in developed and developing countries to look at the impact of education on behavior change. In New York City, a single session education program delivered through a mobile mammography unit targeting immigrant and refugee women in the area led to significant improvements in breast cancer knowledge and mammography completion. The study highlighted the importance of designing such programs in community-based settings to show social support as well as the need to have health literate and culturally competent interpreters as part of the team to ensure an accurate and successful communication process (8). Another quasi-experimental study based on the Health Belief Model was implemented in Iran to look at the effect of education on the behavior of breast cancer screening in women. The pre- and post-tests administered in the form of a researcher-made questionnaire three months before and after four teaching sessions showed remarkable improvements in knowledge, perceived severity, perceived benefits, perceived barriers, and cues to action (10). Both descriptive and experimental studies showed consistency in personal and environmental determinants. The identified determinants will be targeted in the following intervention plan at four different levels of the socioecological model: intrapersonal (refugee women); interpersonal (physicians), organizational (UNHCR), and societal (UNHCR & MOPH).

### Delivery Vehicle Preferences for Educational Intervention Components based on Usability of Technological Devices Among Syrian Refugees

Shioiri-Clark (43), a design innovation manager for the International Rescue Committee (IRC), shared her experience when trying to develop an application to promote healthy child development among Syrian refugees in Jordan and Lebanon. Nearly every household in camps had access to at least one smartphone since it is the only communication method refugees are able to use to contact their family members in Syria (33). However, when the IRC team tried to deliver digital health-promoting activities and messages through Whats app, Facebook, and an Android mobile application that Syrian refugee parents had to download, only the former two methods were effective. When pilot testing the app, it was seen that many parents only used their phones to satisfy specific functions. They had no interest in downloading an app to use for one particular purpose and that would take time for them to learn how to successfully navigate. The majority of Syrian refugees used their phones for phone calls or for news updates via Facebook. Moreover, one of the barriers to focusing the intervention on an app was the low literacy level of the targeted parents. However, when health promoting videos teaching parents how to carry out activities to improve their children's health status were uploaded on Whats app groups and Facebook, parents were really excited to carry out the activities since they connected with other refugees featured in the video and were also themselves featured in future videos to share their own success stories. An additional positive outcome resulting from this intervention was the new connections formed between different households as they all supported each other to improve their children's health (43). One of the main components that will be used to deliver health messages to refugee women and their families is an awareness and action-oriented video. The delivery vehicles for this video are the two mobile applications “Whats app” and “Facebook” rather than TVs or radios since satellite connection might be limited in camps (further information in step 4).

### IM Step 2: Program Objectives

After conducting the needs assessment based on a thorough review of the literature, the overall behavioral outcome was defined as “Syrian and Iraqi refugee women residing in camps located in Beirut, Lebanon will perform a mammogram once a year if aged between 30 and 55 and once every 2 years if aged 55 and above. The age range mentioned is different than that set by the American Cancer Society (annual mammograms for women aged between 40 and 54 and biennial mammograms for women aged 55 and above) since the age standardized incidence rate for breast cancer at diagnosis among Arab women in the MENA region (usually 27–30 years old and above) was one fourth to one third that of Western women (44). Hence, screening at an earlier age for breast cancer is recommended in Lebanon (45). Mammography plays a crucial role in decreasing metastatic breast cancer incidence and prevalence rates by detecting the disease at an earlier and less advanced stage compared to clinical diagnosis. For instance, the study carried out by Ekeh et al. (46) showed that low-income patients were more likely to respond positively to treatment when diagnosed with stage II carcinoma by a mammography evaluation compared to individuals whose breast cancer was detected clinically at a more aggressive stage of the disease. Furthermore, increased uptake of mammography was identified as the underlying reason for the improved cancer-related morbidity and mortality rates reported in the U.S over the past two decades. This assumption was further supported by the American Cancer Society, which highlighted a 34% decrease in breast cancer death rates between 1990 and 2010 due to the widespread availability of screening measures across the country (47). One of the main performance objectives was having Syrian and Iraqi refugee women in Lebanon perform self-examination for early detection of nodules starting at age 25. The fulfillment of suitable self-examination procedures can contribute to the early detection of the disease. Over 40% of verified breast cancer cases were detected by the patient primarily after reporting to their physician that a lump or nodule was forming in their upper chest in either both or one of their breasts (48). This behavioral outcome is also related to the BHGI resource stratification guidelines which integrate awareness, breast self-examination (BSE), clinical breast examination (CBE), and mammography as primary modalities for the early diagnosis of the disease in low-income countries. Increasing knowledge among the target population along with BSE and CBE measures can reduce the burden of late stage breast cancer detection (3, 20).

As for the primary environmental outcome, it aims to increase UNHCR support for refugee women to receive early detection (screening and self-examination) and treatment (chemotherapy, radiology) measures (organizational level). Refugee women are constantly struggling to access quality treatment at an affordable rate, especially those residing in camps located in developing countries such as Lebanon, since these nations themselves are incapable of meeting the healthcare needs of their own citizens. El Saghir et al. (3) reported that the UNHCR office in Lebanon is facing an 83% deficit in funds, particularly in financial and monetary aid allocated to the provision of medical services to refugees. Furthermore, (49) highlighted that restricted access to preventive services due to high medical expenditures, lack of insurance, and poor infrastructure contributed to worsening breast and cervical cancer screening rates in 15 developing countries. Therefore, the assistance of UNHCR through resource-stratified guidelines could aid in decreasing the alarming metastatic breast cancer incidence rates among refugee populations in third world countries. Access to subsidized or free screening procedures in health maintenance organizations and primary healthcare centers increased willingness and conformance to screening recommendations among low-income women compared to those who did not obtain similar advantages and financial help (50).

The secondary environmental outcome will enhance physicians' communication skills with refugee women to emphasize the importance of screening and recommend affordable treatment measures (interpersonal level). A physician-patient relationship that is trustworthy and respectful in nature is a valuable asset in decreasing metastatic breast cancer cases since it enhances the willingness of the targeted population group to regard their doctors as reliable sources of information and conform to the recommended health-actions. Therefore, if the physician allocates only limited time for the purpose of explaining the crucial necessity to conform to the recommended preventive and treatment measures using lay terms or emphasizing the importance of completing the chemotherapy treatment if diagnosed with metastatic breast cancer, vulnerable populations are not likely to perform their annual/biannual mammography and adhere to their prescribed treatments as depicted by international guidelines. Physicians should accommodate their explanation based on the educational level of the refugee women since some women can hold a university degree while others dropped out of school at an earlier age. A study carried out by Ekeh et al. (12) showed that among 625 low-income females in Jordan who had access to free mammography vouchers after successfully participating in home-based breast cancer awareness sessions led by health literate and culturally competent health experts, 73% were reported to seek screening services, mainly mammography, in nearby primary health centers. Knowledge about the vitality of screening and self-examination in enhancing treatment and recovery options was seen to improve significantly as a result of the fulfillment of sequential awareness and communication sessions between the target population and the team of health professionals. Follow-up visits increased compliance to the recommended prevention measures (12). El Saghir et al. (19) emphasized the importance of the role of the primary care providers in responding to the healthcare burden brought upon an increasing cancer population in low- and middle-income countries. By developing training curricula in cancer etiology, prevention, and early detection, along with improving communication skills with the disadvantaged population, increased effectiveness of care provided to the patients will be attained (19).

The tertiary environmental outcome will entail a collaboration between UNHCR and the Lebanese MOPH to work on formulating a policy to subsidize by 75% chemotherapy fees for refugee women in dire need of treatment. According to the basic and limited levels of the BHGI guidelines, preoperative chemotherapy with AC (Adriamycin and Cytoxan), EC (Epirubicin and cyclophosphamide), FAC (fluorouracil, doxorubicin, and cyclophosphamide) or CMF (cyclophosphamide, methotrexate, and fluorouracil) should be offered to breast cancer patients in low-resource settings with a minimum of 75% reach and a target of 90% (3, 20). However, the lack of policies in the country to protect the basic human rights of refugees such as accessing healthcare services at a reasonable price in the country's medical establishments renders it nearly impossible to treat chronic diseases such as breast cancer (17, 18). Therefore, the Lebanese Ministry of Public Health should join efforts with UNHCR and advocate for the implementation of a policy which increases the allocation of funds for breast cancer chemotherapy that should be mainly provided by the Ministry which acts as the primary healthcare reference for the target population (50% of funds) and supplemented by the international agency (25% of funds). This will allow the UNHCR to focus on diverse health and social issues affecting the refugee population in Lebanon. According to a global policy analysis of resource-stratified metastatic breast cancer policy development conducted across 16 countries having diverse geographic areas, socioeconomic statuses, and healthcare systems, the implementation of policies was successful in the adoption of national cancer control programs for metastatic breast cancer. However, gaps including absence of specialized physicians, inadequate public awareness, lack of efficient care delivery, and limited accessibility to required treatment highlight the need for advocacy efforts and promising models to support policy adoption and widespread adaptation across the key health sectors in these countries. Holistic interventions are needed that tackle several levels of the socioecological model at once to ensure long-term sustainability of programs (51).

### Performance Objectives and Change Objectives

Performance objectives (POs) are developed by program planners for behavioral and environmental outcomes to ensure that the participants are performing at a criterion level that enhances the success of the developed intervention (21). The anticipated behavioral and environmental outcomes were further subdivided into POs. Matrices of change objectives (COs) were formulated for each of the identified behavioral and environmental outcomes. COs are considered to operate as a blueprint of the theoretical design rationale since they act as active ingredients of the intervention (52). Tables 1A–D list the POs of the different agents involved in this intervention (refugee women, physicians, UNHCR, and MOPH) along with their respective change objectives.

**Table 1A:** Performance Objectives for the Individual Behavioral Outcome: Syrian and Iraqi refugee women in Lebanon will undergo a mammogram once a year if aged between 30 and 55 and once every 2 years if aged 55 and above.

**Performance objectives**	**Determinants**			
	**Self-efficacy**	**Knowledge**	**Perceived susceptibility**	**Perceived barriers**
PO1: Iraqi and Syrian refugee women will communicate with physicians about their fears and learn about the benefits of screening to correct any misconceptions	S1: Express confidence in their ability to share and overcome their fears S2: Express confidence in their ability to learn about benefits of screening	K1: State the consequences of not screening at the recommended age K2: Recognize that performing a yearly/biannual mammography can reduce your risk of metastatic breast cancer and its complications, benefits of early detection	PS1: Recognize that they need to screen starting at the age of 30 as they are susceptible to breast cancer	PB1: Demonstrate understanding about the benefits of screening by correcting their misconceptions PB2: Recognize that preventive measures such as screening do not interfere with and disrespect religious beliefs
PO2: Iraqi and Syrian refugee women will plan to have annual or biennial mammogram based on the international guidelines for screening	S1: Express confidence in their ability to get screened at the appropriate age for screening	K1: Demonstrate understanding of the relationship between age of screening recommended to risk factors, genetic factors, family history, and environmental factors K2: Differentiate between annual and biennial screening measures	PS1: Explain that they are more susceptible to breast cancer at an early age due to different environmental, genetic, and social stressors	PB1: Identify nearby hospitals and clinics that offer mammograms to set an appointment
PO3: Iraqi and Syrian refugee women will go through free governmental buses to hospitals, mobile clinics, and primary healthcare centers to access the needed care (screening and mammography)	S1: Express confidence in their ability to utilize free transportation means provided by the government to access screening and treatment services	K1: List the different hospitals and PHCs that are part of the intervention K2: State the different services they have access to and their point of contact for each service (radiologist: screening/ oncologist, registered nurse: chemotherapy) K3: Describe where to go (bus stops) to ride the designated free buses		PB1: Recognize that barriers to accessing screening and chemotherapy related to distance are resolved through free transportation
PO4: Iraqi and Syrian refugee women will follow up with their healthcare providers 10 days after screening to discuss results	S1: Express confidence in their ability to follow up with their healthcare provider 10 days after mammography to discuss their results S2: Express confidence in seeking treatment if diagnosed previously or newly with breast cancer	K1: List the steps of successful follow-up K2: Identify ways to communicate with the healthcare provider before and after follow-up for screening	PS1: Demonstrate understanding of cancer relapse or worsening if proper follow-up is not carried out PS2: Explain that follow-up is important for screening to address tumors detected at an early stage and prevent progression into metastatic stages	PB1: Recognize that healthcare providers in partner institutions are willing to care for the patient on a long-term basis PB2: Explain that transportation and follow-up fees will be covered by UNHCR and partner local institutions (hospitals, MOPH, NGOs)
PO5: Iraqi and Syrian refugee women, who are diagnosed with breast cancer, will follow up with their healthcare providers once every week/month or every other week to receive the needed chemotherapy	S1: Demonstrate the ability to follow up with the healthcare provider based on a schedule set by the physician	K1: Identify ways to communicate with the healthcare provider before and after follow-up for chemotherapy	PS1: Demonstrate understanding of cancer relapse or worsening if proper follow-up is not carried out PS2: Demonstrate understanding of importance of adherence to chemotherapy regimen to avoid relapse or deteriorating conditions	PB1: Recognize that healthcare providers in partner institutions are willing to discuss and check response to the treatment to ensure compatibility and effectiveness
PO6: Iraqi and Syrian refugee women will set up an appointment with their healthcare provider once treatment is completed to ensure proper follow-up	S1: Demonstrate the ability to set up an appointment with the healthcare provider once treatment is terminated	K1: Explain the need to follow-up with the healthcare provider once treatment is terminated to examine treatment progress and side-effects	PS1: Demonstrate understanding that physician check-up is needed since chemotherapy can adversely affect other organs PS2: Recognize that additional medications might be prescribed to avoid relapse and to decrease infection and debilitation rates	PB1: Recognize that healthcare providers in partner institutions are willing to follow-up with the refugee women to ensure full recovery
PO7: Iraqi and Syrian refugee women will delegate childcare and chores during the assigned screening/chemotherapy time by asking the help of their elderly neighbors, family members, and young adults (women aged less than 30)	S1: Demonstrate confidence in resolving childcare and household chores by seeking help of neighbors, family, and friends during screening/treatment time	K1: Identify people in the community who can be trusted with childcare K2: Describe steps to make an agreement about childcare with partner and family members		PB1: Recognize that close family and friends are capable of support in time of need PB2: Recognize that children can be left on their own for a few hours under adult supervision PB3: Recognize that screening/treatment is more important than chores
PO8: Iraqi and Syrian refugee women who were confirmed free of breast cancer should perform monthly (3-5 days after menstrual cycle) breast self-examinations	S1: Demonstrate confidence in the ability to apply proper self-examination methods	K1: Describe steps to self-examination K2: Identify the correct way for detecting nodules		PB1: Recognize that healthcare providers will teach them an easy yet accurate way of early detection of nodules

**Table 1B:** Performance Objectives for the Interpersonal Environmental Outcome: Physicians communicate with refugee women about importance of screening and recommend affordable treatment measures.

**Performance objectives**	**Determinants**			
	**Self-efficacy**	**Knowledge**	**Perceived barriers**	**Perceived severity**
PO1: Physicians will collaborate with expert interpreters in delivering sensitive health messages to refugee women about their cancer status	S1: Express confidence in the ability to collaborate with translators and interpreters to deliver culturally sensitive accurate health messages	K1: State the consequences of not communicating the correct health messages to refugee women K2: Identify ways to simplify the delivery of health messages (pictures, videos, role playing)	PB1: Express willingness to use communication skills for effective delivery of health services (screening and chemotherapy)	PS1: Demonstrate understanding of the healthcare burden brought on by late screening and cancer detection PS2: Identify the severe social, economic, and health consequences of inappropriately addressing metastatic cancer due to language and cultural barriers
PO2: Physicians will explain to refugee women how screening and treatment procedures are carried out to appease fears and concerns	S1: Demonstrate the ability to effectively explain screening and treatment procedures using lay terms S2: Express confidence in the ability to appease fear and concerns by addressing misconceptions through the help of interpreters	K1: State that the majority of refugee women are illiterate and are unaware of breast cancer prevention measures (screening) K2: State that Syrian and Iraqi refugee women will fail to screen for or treat breast cancer without a full understanding of the severity of the disease and the positive impact of screening (early) and uninterrupted chemotherapy sessions (late) K3: Explain that fears and concerns must be addressed to have a successful intervention	PB1: Plan different methods to ensure the successful receival of health messages PB2: Explain in lay terms the importance of early breast cancer detection PB3: Assure refugee women that no pain will be felt when performing a mammography PB4: Assure refugee women that screening fees will be covered	PS1: Explain the severity of metastatic cancer and its long-term burden on their overall health and economic status
PO3: Physicians will answer questions about breast cancer, screening, and chemotherapy in lay terms	S1: Express confidence in the ability to build trustworthy relationships by encouraging refugee women to ask questions and share thoughts	K1: List the benefits of early breast cancer detection K2: List the consequences of interrupted chemotherapy treatment	PB1: Repeat health-related information more than once to ensure understanding by all refugee women	PS1: Recognize that miscommunication could inflict harm upon the refugee women in terms of understanding the severity of the disease and the need to take action
PO4: Physicians will sign orders for refugee women to receive free or significantly subsidized screening and treatment through referrals	S1: Express confidence in the ability to refer refugee women to partner hospitals and health centers for mammography and treatment	K1: List all hospitals and primary healthcare centers providing free or subsidized treatment to refugee women	PB1: Explain that all screening and treatment services will be fully covered by UNHCR and other referral hospitals and health centers	PS1: Recognize that assurance of free or minimized fees for screening and treatment measures is necessary to increase screening and treatment rates among refugee women
PO5: Physicians will encourage women to screen once every year or every other year depending on age, family history, and risk factors	S1: Express confidence in the ability to encourage women to screen based on recommended guidelines S2: Express confidence in the ability to explain to refugees why (risk factors) they need to start screening at a specific age	K1: State and explain to refugee women the benefits of screening at the recommended age K2: List the different risk factors that influence the age of onset of breast cancer	PB1: Organize a mammography/ treatment plan for each of the refugee women to avoid confusion or omission PB2: Ensure that mammography will be provided for free in the intervention settings (partner public hospitals and PHCs)	PS1: Explain the role of early self-examination in decreasing the severity of late stage breast cancer complications

**Table 1C:** Performance Objectives for the Organizational Environmental Outcome: UNHCR will support refugee women in receiving early detection (screening and self-examination) and treatment (chemotherapy) measures.

**Performance objectives**	**Determinants**			
	**Self-efficacy**	**Knowledge**	**Perceived barriers**	**Perceived severity**
PO1: UNHCR Executive Office will increase overall funding for chronic disease management (breast cancer) among Syrian and Iraqi refugee women in its Regional Bureau in Lebanon	S1: Demonstrate the ability to allocate the necessary funds for chronic diseases while maintaining needed funds for infectious diseases	K1: State the short-term consequences of not funding cancer screening and treatment K2: State the long-term consequences of not funding cancer screening and treatment	PB1: Express willingness to increase funds to manage chronic diseases by reducing funds allocated to less urgent issues (infectious diseases that have been eradicated)	PS1: Demonstrate understanding of the healthcare burden brought by late screening and cancer detection PS2: Identify the severe social, economic, and health consequences of metastatic cancer
PO2: UNHCR Public Health Division in Lebanon will collaborate with the EU-funded European Neighborhood instrument, UNDP (United Nations Development Program), WHO, local NGOs, and the Ministry of Public Health to cover the remaining 25% of screening and treatment costs for Syrian and Iraqi refugee women	S1: Demonstrate the ability to collaborate and coordinate efforts with the public and private sector at the local and international levels S2: Express confidence in the ability to effectively allocate funds for screening and treatment purposes at the hospitals and primary healthcare centers involved in the intervention.	K1: Express the need to unite efforts and form public-private partnerships for a successful intervention K2: Understand that Syrian and Iraqi refugee women will fail to screen for or treat breast cancer without the coverage of the remaining 25% of the health services fees.	PB1: Organize several meetings to overcome any unexpected and expected challenges between the different parties PB2: Plan monthly financial meetings to discuss budget allocation and prevent any outfalls PB3: Propose new local and international partnerships to increase overall funding for breast cancer management	PS1: Maintain continuous coverage of 25% for screening and chemotherapy to avoid severe comorbidities associated with metastatic cancer PS2: Explain the severity of metastatic cancer and its long-term burden on the healthcare system PS3: Emphasize the continuous need for funding to decrease the alarming incidence and prevalence rates of breast cancer in Lebanon
PO3: UNHCR Department of Education will collaborate with the Ministry of Higher Education and UNESCO (United Nations Education, Scientific, and Cultural Organization) to provide culturally competent and health literate interpreters and translators to assist health professionals in delivering accurate health messages to refugee patients	S1: Express confidence in the ability to provide refugee women with health literate and culturally competent interpreters when accessing the needed care S2: Demonstrate confidence in the ability to create a successful collaboration between health professionals and interpreters	K1: Describe how qualified interpreters and translators will facilitate the effective communication process between the refugee women and the healthcare provider K2: List the strategies that interpreters and healthcare professionals will implement to ensure recipient of accurate health messages	PB1: Recognize that accurate interpretation and translation of health messages is essential to deliver a successful intervention PB2: Recognize the need to continuously train the health literacy team to be able to deal with multiple cultural challenges PB3: Expect that the coordination of effort between physicians and interpreters will increase screening and chemotherapy rates	PS1: Recognize that miscommunication could inflict harm upon the refugee women in terms of understanding the severity of the disease and the need to take action PS2: Explain the critical role of health literate and culturally competent interpreters in increasing adherence to screening guidelines and chemotherapy recommendations
PO4: UNHCR Department of Education will collaborate with the Ministry of Higher Education and UNESCO (United Nations Education, Scientific, and Cultural Organization) to provide culturally competent and health literate educational sessions on self-examination and early nodule detection among refugee women aged 25 to 50.	S1: Express confidence in the ability to organize awareness sessions for refugees on breast cancer screening and self-examination S2: Express confidence in the ability to teach refugees appropriate self-examination methods in a private, safe, and accessible environment	K1: State and explain to refugee women the benefits of self-examination K2: Describe how to perform self-examination using videos, pictures, and real-life examples K3: Demonstrate how to feel and detect nodules	PB1: Organize the awareness session in a place where refugee women feel safe and confident in learning about self-examination PB2: Illustrate step-by-step how to carry out self-examination PB3: Discuss all health-related information accurately with the help of qualified translators	PS1: Explain the role of early self-examination in decreasing the severity of late stage breast cancer complications
PO5: UNHCR Department of Support to Host Communities will collaborate with the Ministry of Transport to provide free buses to the health institutions involved in the intervention (at least twice a month for screening and once a week for treatment)	S1: Express confidence in the ability to collaborate with the Ministry of Transport to provide free buses to access the needed care in partner hospitals and clinics S2: Express confidence in the ability to have multiple bus stops to accommodate the needs of all refugee women and avoid distance issues	K1: Explain that transportation services are needed at multiple times of the day to accommodate refugee women K2: Plan a schedule with the Ministry of Transport for bus timing depending on hospital and clinic opening hours	PB1: List and repeat all bus stops for refugee women PB2: Send in-person or paper reminders PB3: Modify bus schedule depending on women's preferences	
PO6: UNHCR Department of Support to Host Communities and the Ministry of Transport will collaborate with physicians in partnering health institutions to set schedules for screening and chemotherapy follow-ups	S1: Express confidence in the ability to collaborate with the healthcare providers in partnering health institutions to set bus schedules for follow-ups S2: Express confidence to include multiple dates for follow-ups to accommodate the needs of all refugee women	K1: Plan a schedule with the physicians depending on shifts and availability K2: Explain that multiple appointment time periods are needed to accommodate the large number of refugee women	PB1: List and repeat all screening/ chemotherapy appointments for refugee women PB2: Send in-person or paper reminders for physicians to ensure continuous collaboration and willingness to allocate time for screening and chemotherapy sessions PB3: Modify bus schedule/ clinic schedule depending on both physician and women preferences	

**Table 1D:** Performance Objectives for the Societal Environmental Outcome: The Ministry of Public Health (MOPH) will formulate a policy that provides full coverage of screening services and 75% coverage of chemotherapy treatment for Syrian and Iraqi refugee women with breast cancer in Beirut, Lebanon.

**Performance objectives**	**Determinants**			
	**Self-efficacy**	**Knowledge**	**Perceived barriers**	**Perceived severity**
PO1: The MOPH General Directorate Department of Control on autonomous public hospitals will formulate a policy in collaboration with UNHCR Public Health Division in Lebanon which renders the MOPH accountable for all screening costs and 50% of treatment costs for refugee women in public hospitals while UNHCR will cover an additional 25% to ensure provision of subsidized treatment	S1: Demonstrate the ability to formulate a policy that protects the rights of refugee women in Lebanon to access affordable chemotherapy treatment in public hospitals and PHCs in Beirut S2: Demonstrate the ability to collaborate with UNHCR Public Health Division to divide financial responsibilities among the different departments	K1: Express the need to unite efforts and form public-private partnerships for a successful policy implementation K2: Understand that the lack of a funding policy that covers chemotherapy and screening fees for refugee women in public hospitals and PHCs of the host community will fail to prevent or treat metastatic breast cancer	PB1: Organize several meetings to overcome any unexpected and expected challenges that might impede the implementation of the policy PB2: Plan monthly financial meetings to ensure that funds are in place for an effective policy implementation process PB3: Propose new local and international partnerships to increase overall funding for breast cancer management and for widespread adoption of the policy at a national level	PS1: Demonstrate understanding of the urgent need of such policy to ensure continuous coverage of screening fees and 50% of chemotherapy fees to avoid the acceleration of the healthcare burden brought upon the host community PS2: Emphasize the need to continuously monitor the adoption of the policy to decrease the alarming incidence and prevalence rates of breast cancer in Lebanon
PO2: UNHCR Public Health Division in Lebanon will collaborate with the EU-funded European Neighborhood instrument, UNDP (United Nations Development Program), WHO, local NGOs, and the Ministry of Public Health to cover the remaining 25% of screening and treatment costs for Syrian and Iraqi refugee women	S1: Express confidence in the ability to incorporate the free mammography portion of the annual national breast cancer campaign which takes place over a period of 3 months as part of the long-term intervention S2: Express confidence to extend free screening services throughout the duration of the entire year for refugee women in Beirut	K1: State and explain which hospitals and PHCs offer free screening services to inform participating medical institutions about the policy K2: Understand that more equipment and human resources are needed for successful policy implementation which requires collaboration with medical equipment companies and academic institutions	PB1: Recognize that merging the free mammography service portion of the National Breast Cancer Campaign in public hospitals and PHCs throughout the duration of the year is essential for the successful implementation of the policy	PS1: Explain the critical role that public hospitals and PHCs play in the delivery of the National Breast Cancer Campaign and the need to collaborate with them for the maintenance of policy adoption
PO3: The MOPH Directorates of Medical Care and Preventive Care will unite efforts with UNHCR Executive Office to monitor adequate allocation of funding for screening and treatment services	S1: Demonstrate the ability to monitor adequate allocation of funding to cover screening and treatment fees for Iraqi and Syrian refugee women in Beirut, Lebanon as stated in the formulated policy	K1: Describe how qualified financial and accounting managers working in both the MOPH and UNHCR will process funds to the designated public hospitals and PHCs K2: Describe how qualified MOPH inspectors will monitor the allocation of funds in participating medical institutions	PB1: Recognize the need to continuously monitor the financial process of receiving and using funds in hospitals to ensure compatibility with policy PB2: Expect that the coordination of efforts between MOPH, UNHCR, and hospital employees will improve adaptation of policy over the long-run	PS1: Recognize that the lack of adequate monitoring and evaluation of financial and monetary processes could result in termination of policy which in turn could inflict harm upon the refugee women in terms of inability to afford treatment measures PS2: Explain the critical role of inspectors and financial and accounting managers in increasing compliance to the formulated policy

### IM Step 3: Program Design

The initial task of step 3 entails brainstorming ideas for a program theme, major intervention components, scope, and sequence. The end product is a draft plan that outlines the anticipated multilevel intervention.

#### Program Components

The main barriers to mammography screening and accessing quality chemotherapy treatment among Syrian and Iraqi refugee women with breast cancer in Lebanon are mainly financial, social, and educational. El Saghir et al. (3) and (1) emphasized the need to increase monetary aid allocated for chronic diseases, patient education, education of representatives of international organizations about the burden of breast cancer and its impact on this vulnerable population, assurance of consistent physician consultations and follow-up, along with aligning referrals with subsidized medical services with the purpose of observing positive significant changes in mammography rates and proper treatment adherence and chemotherapy completion. Hence, the long-term sustainability and success of the intervention in attaining the desired behavior change is dependent on several levels of the socioecological model including the intrapersonal (refugee women), interpersonal (physicians), organizational (UNHCR), and societal (MOPH) levels. At the individual behavioral level, Syrian and Iraqi refugee women residing in camps in the Beirut District of Lebanon will participate in an initial awareness campaign designed to promote the self-management of screening and treatment behaviors to help women overcome their fears and correct their misconceptions which prevent them from engaging in essential health-related actions (1, 3, 8, 15). The campaign will be followed by educational sessions on breast self-examination using role modeling techniques, in addition to informational sessions that will address the multiple ways refugee women can access the required care at the participating hospitals and primary health centers through free governmental transportation measures based on a previously set bus schedule that satisfies the preferences of both the refugee women and the physicians (53). Both types of sessions will be organized within the refugee camp setting and delivered by primary care physicians and specialized oncologists with the help of public health workers, community leaders, and interpreters and translators to assure the transmission of clear and culturally competent health and logistics information (54). An emphasis on the value of follow-up sessions and its powerful impact on the recovery process will be incorporated in the educational sessions. Refugee women will also be provided the opportunity to ask the on-site healthcare experts and educators about any concerns or uncertainties they have regarding their health status or the information they obtained throughout the progress of the program (1, 8, 15, 19, 46, 47, 47).

Environmental restructuring is a vital intervention component that needs to be achieved to ensure that the targeted behavior change will be maintained. At the physician level, change will consist of mandatory online trainings for physicians and public health workers to acquire culturally competent and health literate ways to effectively and clearly communicate with refugee women about the importance of undertaking mammography and completing their chemotherapy treatment. The online version of the trainings is more cost-effective and more feasible to physicians as they can complete the required sessions depending on their availability. Follow-up appointments will be integrated as part of a social support system for all refugee women to allow physicians to address re-emerging concerns and worries after screening or chemotherapy sessions (1, 12, 15, 19, 55).

At the organizational and societal levels, change will encompass the introduction of a new budget plan that calls for an increase in annual funds allotted to the management of chronic diseases. The new financial plan will be settled once the administrators and coordinators of several UNHCR-based departments and MOPH departments have arranged meetings with hospital board of directors, governmental representatives, and physicians working in participating public hospitals and PHCs to discuss the refugee camp environment. They will review findings from surveys completed within the refugee camps as well as the guidelines and protocols that should be developed for change to ensure that a collaborative approach has been adopted to establish a final budget plan that satisfies all involved sectors. The surveys, conducted by the planning group, will assess the refugee camp environment in terms of available resources, influential leaders in the camps, readiness of refugee women to participate in the intervention, along with potential barriers that might be encountered. This will help to identify any urgent barriers that need to be addressed prior to program implementation. The final budget plan will act as the foundation of the newly formulated policy which holds the MOPH fully accountable for screening services and holds both the MOPH (50%) and UNHCR (25%) accountable for the provision of subsidized chemotherapy fees as recommended by the resource-stratified guidelines (3, 20). The organizational strategic approach to change which incorporates leadership skills, future goals, and effective collaboration is considered to be an evidence-based practice for long-term change (56, 57). To raise awareness of the severity of metastatic breast cancer among refugee women in Lebanon and highlight the necessity of targeting the underlying and interfering factors (restricted funding, lack of awareness, limited access to quality treatment, no proper follow-up measures, fear), UNHCR and MOPH representatives will be invited to attend informational and orientation sessions prepared by a team of oncologists, physicians, community leaders, public health workers, and interpreters to fortify understanding of the obligation to deal with this particular health issue (1, 15, 16, 19, 30, 49, 50, 55, 58).

Newspapers, Public Service Announcements (PSAs), billboards, flyers, radio, and TV channels will be adopted as reinforcing channels and vehicles to increase attainment of the positive health behaviors. These media tools will be used to inform refugee women about the opportunity to participate in a multi-level intervention that will enable them to access the needed medical and preventive services either for free or at a significantly subsidized prize (for chronic chemotherapy treatment). The widespread promotion of the intervention will lead to the desired health outcomes, along with an accurate delivery of direct messages that are tailored to account for cultural and linguistic barriers (21). The mentioned methods will be used due to their success in reaching the Lebanese population during the annual breast cancer campaigns. The WHO has been supporting the annual national breast cancer campaigns since 2008 due to their remarkable success and their effective outreach efforts which has increased by more than 60% since the initial launch of the campaign (59). In 2014, one of the main barriers identified for the lack of interest of some women in seeking a mammography was the lack of support of family members. Therefore, the media campaign (flyers, videos, and billboards) was modified to portray women with their children and husbands to encourage family members to remind their wives/aunts/mothers to make a screening appointment. In 2016, fear was identified as an additional factor affecting screening rates among women. This lead the MOPH health promotion experts to think about creative outlets that could grab the attention of the Lebanese population at large. The Breast Cancer Pink Ribbon was then made in Lebanon's largest sports stadium using over 8,000 pink balls and was recorded in the World Academy record as the largest ribbon ever created (60, 61). A rise from 9,879 completed screenings in 2016 to a total of 21,752 was attributed to the success of the campaign which empowered women and their families to fight against breast cancer by taking the necessary preventive measures (37). By adapting a similar information environment and tailoring it to account for the culture of refugee women, the behavioral outcome is more likely to be successfully attained (54).

#### Program Theme

The program's theme will focus on empowering refugee women to screen for breast cancer and to seek treatment as recommended by their physicians. “My Right, My Fight” (Arabic- 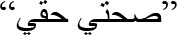) is the chosen slogan for the intervention. “My Right” refers to the fact that Lebanon has no policy protecting the basic human rights of refugees such as accessing healthcare services at a reasonable price in the country's medical establishments (17). “My Fight” has to do with the actual empowerment act, which aims to encourage women to perform annual mammography and complete their chemotherapy treatment fully, if needed.

#### Program Scope and Sequence

Table 2 provides a general overview of the program's intended scope and sequence. Note that the scope and sequence of the intervention might be modified depending on the willingness of the politicians to cooperate and the overall stability of the country. All activity time periods will be adjusted accordingly.

**Table 2:** Scope and Sequence in Months (Note that some components might take longer than expected due to political tension and instability in the country).

**Program components**	**Months 1-3**	**Months 4-6**	**Months 6-12**	**Month 12**	**Months 12-onwards**
Societal Level (MOPH)	Draft of breast cancer funding policy for refugees with the different MOPH departments and UNHCR funding department Meetings with MOPH health promotion experts to incorporate media strategies into the intervention to promote the policy Meetings with the National Breast Cancer Campaign organizing committee to incorporate the free mammography services as a part of the year-long intervention Meetings with advisory committee and planning group to get feedback on policy draft Involve legal people in the process Meetings with sponsors to help in coverage of fees to ensure sustainability of policy	Work on getting support from the parliament members and from ministers to ensure enough votes for the policy to pass Breast cancer educational sessions for different political parties and ministries to broaden support at a national scale	Passing of policy draft to the house of deputies in legislative branch Continuous advocacy efforts by UNHCR and MOPH to ensure enough number of votes Get student volunteers to spread news and join human rights issue Continuous press releases to inform the public and get support at a national level	Follow-up on policy execution Formation of partnerships with local and international organizations for sponsorship purposes	Follow-up on policy execution Ensure implementation within the 2-year deadline Continuous formation of partnerships with local and international organizations for sponsorship purposes
Organizational Level (UNHCR)	Survey of refugee camp environment Change planning meetings with advisory committee/planning group Guideline and protocol development with translators, interpreters, and local NGOs Orientation meetings with hospital medical staff	Report on environmental survey Set finalized budget Set finalized schedule for screening and treatment services at participating hospitals Training meeting for health and culturally competent translators and interpreters Placement of billboards and PSAs Newspaper stories and news releases introducing the program and objectives, coverage of the screening facilities, and treatment options available at multiple PHCs and public hospitals	Review training meetings for health providers and trained interpreters Introduce the awareness campaign to the refugee women Set bus schedule and bus stops in collaboration with the Ministry of Transport	Finalize bus schedule based on refugee women preferences (back and forth from hospitals to refugee camps) Update budget plan along the implementation process	Continuous funding opportunities for chronic disease management (breast cancer) among refugee women Updated twice a year about incidence and prevalence rates from physicians to evaluate the impact of the intervention Modify PSAs and billboards to include real survivorship stories
Interpersonal Level (Healthcare providers)		Training sessions with culturally competent translators and interpreters Set schedule in collaboration with hospital board to provide free screening and subsidized treatment measures for refugees	Raising awareness about screening and treatment Conduct educational sessions on self-examination Answer questions and concerns regarding screening and treatment	Encourage women to perform mammography for free Explain the importance of adhering to the right treatment using recommended cycle Set up a health action plan for each individual woman	Follow-up for all refugee women (Influx is not expected to increase significantly due to saturation of camps) Set up the follow-up appointments on screening and chemotherapy results Continuation of treatment Monthly routine check-ups
Individual Level (Iraqi and Syrian refugee women residing in camps in Beirut District, Lebanon)			Get involved in educational sessions Learn about screening and chemotherapy	Set appointments for mammography and/or chemotherapy at one of the participating hospitals or PHCs	Receive treatment and complete follow-up measures as necessary Act as a role model for other refugee women in similar camps

#### Theoretical Methods and Practical Applications

According to Bartholomew Eldredge et al. (21), theory-based change methods are general procedures aiming to influence determinants of behavioral and environmental outcomes. As for practical applications, they consist of real-life examples of how these methods will be delivered to the target population. However, researchers should make sure that these applications are culturally relevant to the context in which the intervention takes place (62). Parameters for use provide guidance to the health promotion program planners when applying these change methods by helping them take into account the diverse characteristics of the environment they are targeting (21).

Change methods at the individual level include active learning, modeling, verbal persuasion, facilitation (Social Cognitive Theory), tailoring, reinforcement, consciousness raising (Transtheoretical model), self-management, and goal-setting (Theories of Self-regulation). The main purpose behind these selected methods is to emphasize the severity of the disease for refugee women while highlighting the importance of screening and chemotherapy adherence in reducing the burden of breast cancer. The methods will be implemented in a supportive environment that eliminates anticipated structural and monetary barriers, in addition to providing hope for the target population by hearing success stories from refugee women survivors. At the interpersonal level, methods include framing (Protection Motivation theory), consciousness raising, self-reevaluation, environmental reevaluation (Transtheoretical model), mobilizing social support (Diffusion of Innovation theory), and personalizing risk (Precaution-Adoption process model). These strategies aim to influence healthcare providers by highlighting the alarming incidence and prevalence rates of breast cancer among the refugee population and stressing their role in improving the burden on the healthcare system through the development of effective health literate and culturally competent communication skills to educate the refugee population about prevention and treatment measures available to them in the country. By advocating for empathy and understanding when dealing with refugee women through the chosen methods, physicians are more likely to allocate their time to provide free mammography, subsidized treatment, and appropriate follow-up as depicted in the intervention plan. For the organizational level, verbal persuasion, setting graded tasks (Social Cognitive Theory), consciousness raising, self-reevaluation, environmental reevaluation (Transtheoretical model), arguments (Communication-Persuasion matrix), and planning coping responses (Attribution Theory and Relapse Prevention Theory) were some of the chosen methods to encourage UNHCR representatives to increase funding for breast cancer chronic disease management and adhere to the policy once implemented. These methods will also increase awareness about the severity of the disease and reduce the perceived barriers that might render them hesitant toward adopting and maintaining implementation of the intervention. Similar methods will be adapted at the societal level to allow the MOPH department representatives to acquire the skills needed to develop an effective policy draft to ensure the sustainability of funding, increase knowledge about the necessity of such a policy to alleviate the burden of breast cancer among refugee women, decrease perceived barriers including the complicated process of passing the policy in parliament, and increase perceived severity of breast cancer among refugee women to enhance motivation to advocate for the execution of the proposed policy. Tables 3A–D display examples of theoretical methods for determinants selected in Step 2 of IM. Parameters for effective implementation were identified for each method (column 3). The following tables can be considered as the blueprint of the intervention where all COs will be covered by the planned program.

**Table 3A:** Selected Methods, Parameters for Use, and Practical Applications for the Determinants of the Behavioral Outcome “Syrian and Iraqi refugee women in Beirut District of Lebanon will undergo a mammogram once a year if aged between 30 and 55 and once every two years if aged 55 and above”.

**Determinants**	**Methods (theory)**	**Parameters for use**	**Practical applications**
Self-efficacy for conducting a breast exam, obtaining a mammogram, and completing treatment if needed	Self-management/Theories of self-regulation	Management of early detection of breast cancer through self-examination, and data collected from healthcare provider about frequency and efficiency of self-examination will be used to reinforce behavior	Guided practice of self-examination of breast nodules in-person and through distributed brochures containing infographic messages
	Goal-setting/Theories of self-regulation	Commitment to goals that are feasible	Requires commitment of refugee women to set goals (screening, self-examination, chemotherapy, follow-up) which could be tracked on the fillable appointment calendars provided to them to avoid recall bias
	Verbal Persuasion/Social Cognitive Theory	Credible source	Educational video prepared in lay language featuring role model refugee women of the same culture who obtained a mammogram and completed their chemotherapy treatment based on physician recommendation
	Reinforcement/Transtheoretical Model	Reinforcement tailored to the individual, to follow behavior in time, and to be seen as a consequence of the behavior	Healthcare provider and family encouragement
	Motivation/Self-determination Theory	Supportive relationship between health professional and refugee women combined with the evocation of patient change talk. Autonomy rather than authority and exploration rather than explanation	Effective communication, collaboration, and confrontation between refugee women and health professionals
Knowledge about proper self-examination, goal achievement, and effective screening and treatment opportunities	Active Learning/Social Cognitive Theory	Time, information, and skills	Encouraging learning from goal-driven and activity-based experience
	Tailoring/Transtheoretical Model & Protection Motivation Theory	Tailoring variables related to behavior change and to culture relevance	Feedback about performance and goal achievement over time through data collected from physicians and UNHCR
	Consciousness Raising/ Health Belief Model and Transtheoretical model	Problem-solving, collective self-efficacy, raising awareness, and changing misconceptions	Self-examination feedback and assistance from health providers in setting up screening and treatment appointments
	Providing Cues to Action/Theories of Information Processing	Cues work best when people are allowed to select and provide their own cues	Affirmation that the information given in the video regarding the covered fees for preventive and treatment services will actually happen in real life as part of intervention
	Elaboration (Theories of Information Processing)	Individuals with high motivation and high cognitive ability; messages that are personally relevant, surprising, repeated, self-pacing, not distracting, easily understandable, and include direct instructions; messages that are not too discrepant and cause anticipation of interaction	Increasing knowledge about the importance of having an annual mammography and correcting any misconceptions related to screening through the video messages
	Self-reevaluation/Transtheoretical Model	Cognitive and affective appraisals of one's preventive efforts; can use feedback and awareness raising followed by problem-solving and increasing self-efficacy	Imagining oneself cancer free or believing in the ability to overcome cancer through effective treatment and follow-up
	Modeling/Social Cognitive Theory	Observational learning, attention remembrance, self-efficacy and skills, identification with model, coping model instead of mastery model	The health provider finds a role model from the community (cancer survivor) who will encourage early detection methods and who will share her coping methods
	Using Imagery/Theory of Information Processing	Familiar physical or verbal images as analogies to a less familiar process	Patient educator helps refugee women memorize self-examination steps by attaching images in a place that is part of a daily routine
Perceived Susceptibility of refugee women toward being prone to a breast cancer diagnosis	Belief Selection/Theory of Planned Behavior and Theory of Reasoned Action	Requires investigation of the current attitudinal, normative, and efficacy beliefs of the individual (not susceptible, cannot do anything to prevent cancer) before choosing the beliefs on which to intervene	The refugee women's belief that they are not susceptible to breast cancer and that no action can lead to early cancer detection should be altered; the value of screening needs to be reinforced; and the belief that screening can help in detecting breast cancer at an early stage needs to be reintroduced
	Persuasive Communication/Communication-Persuasion Matrix and Diffusion of Innovations Theory	Messages need to be culturally relevant and not too discrepant from the beliefs of the individuals	Watching an educational video about the proper way of carrying out self-examination and listening to testimonials from women of the same culture who survived breast cancer due to screening and proper chemotherapy adherence which can significantly influence the perceived susceptibility beliefs of the refugee women
Perceived Barriers toward screening and treatment including literary, financial, and transportation issues	Participation/Diffusion of Innovation Theory	Willingness of refugee women to participate in activities organized by health providers	Participation of refugee women in educational and communication sessions through the help of interpreters
	Individualization/Transtheoretical Model	Personal communication efforts that appease concerns and respond to a learner's needs	Requesting help of interpreters to ask questions about screening, treatment, and funding issues
	Facilitation/Social Cognitive Theory	Identification of barriers and facilitators and the power for making the appropriate changes	Utilization of free buses and free mammography and subsidized treatment in the list of hospitals provided

**Table 3B:** Selected Methods, Parameters for Use, and Practical Applications for the Determinants of the Interpersonal Environmental Outcome “Physicians communicate with refugee women about importance of screening and recommend affordable treatment measures.”

**Determinants**	**Methods (theory)**	**Parameters for use**	**Practical applications**
Self-efficacy of health providers to communicate the benefits of screening and chemotherapy adherence to refugee women in a culturally relevant and health literate way	Framing/Protection motivation theory	Requires high self-efficacy expectations. Gain frames are more readily accepted and prevent defensive reactions	Missing early detection of breast cancer by not getting a mammography every year can further burden the healthcare system (loss frame). Getting a mammography every year lowers the treatment and follow-up burden of millions of refugee women
	Guided Practice; Enactive Mastery experience/ Social cognitive theory	Demonstration, instruction, and enactment; requires willingness to accept feedback	Health literate and culturally competent interpreters and translators will walk healthcare providers through the appropriate way to encourage and promote screening and treatment and then allow healthcare providers to give examples about their expected performance to provide them with the necessary feedback to improve their skills
Knowledge about the seriousness of breast cancer rates among refugee women and the need to take corrective action	Consciousness raising/ Health belief model and transtheoretical model	Raising awareness must be quickly followed by increase in problem-solving ability and self-efficacy skills	Feedback about the continuous increase in the incidence of metastatic breast cancer cases among refugee women due to low screening and treatment rates
	Self-reevaluation/Transtheoretical Model	Stimulating cognitive and affective appraisal for increases in self-efficacy and empathy skills	Empathy training to empathize and understand the challenges that refugee women go through and understand their perceptions toward screening
	Environmental Re-evaluation/Transtheoretical Model	Serving as a role model to others	Educating healthcare providers about how most effectively communicate and approach this vulnerable population
Perceived Barriers toward communicating the health information to the refugee women patients	Planning coping responses/Attribution Theory and Relapse Prevention Theory	Identification of high-risk situations and practice of coping response	Physicians learn how to cope with literacy barriers by communicating the health messages in a culturally competent way using lay terms and by showing willingness to repeat the messages more than once to ensure full comprehension on behalf of the refugee women
	Mobilizing social support/Diffusion of innovation theory	Combines caring, trust, openness, and acceptance with support for behavioral change; positive support is available in the environment	Prompting communication among healthcare providers about benefits of screening and treatment and discussing the facilitated process through the help of UNHCR to make the positive expectations for changing behavior more visible compared to perceived barriers
	Conscious regulation of impulsive stereotyping and prejudice	Not suppressing feelings; conscious self-regulation of automatic stereotyping used effectively	Healthcare providers practice saying “stop thinking this way” as they learn more about Syrian refugees and try to understand their perceptions
Perceived Severity of breast cancer among refugee women at the social, economic, and mental levels	Personalize risk/Precaution-adoption process model	Present messages as individual and undeniable in a culturally competent and health literate way, and compare them with absolute and normative standards that can be understood by refugee women	Physicians understand the long-term economic, social, and health burden of breast cancer among refugee women and learn how to clearly communicate the severity of the health problem to their patients to avoid any misunderstanding
	Arguments/Communication-persuasion matrix	Message new to receiver	Hearing the impact of early detection on decreasing the overall burden of breast cancer among refugee women can influence healthcare providers to encourage screening and provide referrals

**Table 3C:** Selected Methods, Parameters for Use, and Practical Applications for the Determinants of the Organizational Environmental Outcome “UNHCR will support refugee women in receiving early detection (screening and self-examination) and treatment (chemotherapy, radiology) measures.”

**Determinants**	**Methods (theory)**	**Parameters for use**	**Practical applications**
Self-efficacy to set goals and tasks for implementation while maintaining continuous funding	Verbal persuasion/social cognitive theory	Credible source	Representatives from different UNHCR departments view a videotape on the pain and agony felt by Syrian and Iraqi refugee women who die to limited access to treatment and screening. This will be followed by successful breast cancer prevention interventions done in different countries with the same vulnerable population to emphasize the need for increased funding
	Public Commitment/Theories of Automatic, Impulsive, and Habitual Behavior	Needs to be a public announcement; may include contracting	UNHCR signs contracts with public hospitals and PHCs to ensure coverage of screening and chemotherapy services. Contracts will be shared on the morning and evening news to make sure that all refugee women are aware of the newly available healthcare services they could access
	Goal-setting/Theories of Self-regulation	Commitment to the goals despite difficulty	UNHCR, physicians, hospital board members, and involved NGOs discuss the prioritized goals to create a balance between the population need and the external funding and implementing agencies
	Set graded tasks/ Social cognitive theory	Final behavior can be reduced to sub-behaviors	UNHCR divides tasks and funding among all members of the resource group, program adopters, and program implementers. Each group targets a particular sub-behavior with the allocated funds they receive
Knowledge about the challenges that refugee women endure due to limited access to essential healthcare services and the need for continuous funding	Consciousness raising/ Health belief model and transtheoretical model	Raising awareness must be quickly followed by increase in problem-solving ability and self-efficacy skills	Feedback about the continuous increase in the incidence of metastatic breast cancer cases among refugee women due to low screening and treatment rates
	Self-reevaluation/Transtheoretical Model	Stimulating cognitive and affective appraisal for increases in self-efficacy and empathy skills	Empathy training to empathize and understand the challenges that refugee women go through and understand the need for additional funding for the management and treatment of chronic diseases
	Environmental Re-evaluation/Transtheoretical model	Serving as a role model to others	Asking all other international and local agencies to join this humanitarian cause due to its multiple positive impacts on the overall health status of the population
Perceived Barriers toward facilitating access to screening and chemotherapy treatment for refugee women	Planning coping responses/Attribution theory and relapse prevention theory	Identification of high-risk situations and practice of coping response	UNHCR, with the help of the planning group, define the barriers to screening and treatment. Then, solutions are discussed to resolve the identified barriers
	Mobilizing social support/Diffusion of innovation theory	Combines caring, trust, openness, and acceptance with support for behavioral change; positive support is available in the environment	Facilitating access to care through increased funding and prohibiting stigma in participating hospitals related to refugee status as a sign of social support
	Conscious regulation of impulsive stereotyping and prejudice	Not suppressing feelings; conscious self-regulation of automatic stereotyping used effectively	UNHCR representatives affirm that management of chronic diseases has an even higher importance than dealing solely with infectious diseases
Perceived Severity of breast cancer among refugee women through personalization of risk based on environmental and genetic factors	Personalize risk/Precaution-adoption process model	Present messages as individual and undeniable in a culturally competent and health literate way, and compare them with absolute and normative standards that can be understood by refugee women	UNHCR receives personal risk feedback on the breast cancer status of refugee women from physicians, which will help them realize that their stressful lifestyle and genetic make-up predispose them to the disease at a higher rate
	Arguments/Communication-persuasion matrix	Message new to receiver	Hearing the impact of early detection on decreasing the overall burden of breast cancer among refugee women can influence UNHCR to increase funding

**Table 3D:** Selected Methods, Parameters for Use, and Practical Applications for the Determinants of the Societal Environmental Outcome “MOPH will support refugee women in receiving early detection (screening and self-examination) and treatment (chemotherapy, radiology) measures by drafting and implementing a policy which renders the MOPH and UNHCR both accountable to provide the necessary services.”

**Determinants**	**Methods (theory)**	**Parameters for use**	**Practical applications**
Self-efficacy to develop an effective policy that sustains the intervention by rendering the MOPH and UNHCR accountable for funding as required by the resource-stratified guidelines	Verbal persuasion/Social Cognitive theory	Credible source	Representatives from the MOPH departments and from the UNHCR departments will be invited to breast cancer educational sessions organized by public health experts, physicians, and oncologists to highlight the severity of the problem and advocate for the need for a policy which protects the rights of refugees in accessing affordable quality care
	Public commitment/Theories of automatic, impulsive, and habitual behavior	Needs to be a public announcement; may include contracting	The policy will be promoted on all social media outlets, TV channels, and radio stations to get support from the Lebanese community in general, and to inform refugee women that the government cares about their well-being
	Goal-setting/Theories of self-regulation	Commitment to the goals despite difficulty	UNHCR and MOPH departments prioritize goals to ensure that the policy draft will get support from the parliamentary members and ministers to be later on implemented within the 2 year deadline period
Knowledge about the need of such policy to ensure sustainability of intervention and implementation at a broader scope	Consciousness Raising/ health belief model and transtheoretical model	Raising awareness must be quickly followed by increase in problem-solving ability and self-efficacy skills	Feedback about the need to draft and implement a policy due to the continuous increase in the incidence of metastatic breast cancer cases among refugee women as a result of low screening and treatment rates
	Self-reevaluation/Transtheoretical Model	Stimulating cognitive and affective appraisal for increases in self-efficacy and empathy skills	Empathy training to empathize toward the challenges that refugee women go through and understand the need for a policy to maintain funding for the management and treatment of chronic diseases
	Environmental Re-evaluation/Transtheoretical Model	Serving as a role model to others	Asking all other international and local agencies to join this humanitarian cause by helping UNHCR and MOPH in allocating the necessary funds for policy sustainability due to its multiple positive impacts on the overall health status of the population
Perceived barriers toward passing the policy in parliament and getting the support of parliamentary members and ministers	Planning coping responses/Attribution Theory and Relapse Prevention Theory	Identification of high-risk situations and practice of coping response	MOPH department representatives and UNHCR funding and public health department representatives listen to the input from planning group members and legal authority figures to define the barriers to policy execution and implementation
	Mobilizing social support/Diffusion of innovation theory	Combines caring, trust, openness, and acceptance with support for behavioral change; positive support is available in the environment	Gaining social support from governmental sector and non-governmental agencies to further gain the support of the Lebanese community at a national level
	Conscious regulation of impulsive stereotyping and prejudice	Not suppressing feelings; conscious self-regulation of automatic stereotyping used effectively	MOPH department representatives affirm that the implementation of a policy that holds the Lebanese healthcare system accountable for the health of refugee women, particularly chronic disease management, is highly important
Perceived severity of breast cancer among refugee women through personalization of risk based on societal factors such as the lack of a policy which protects the refugees' right to quality and affordable chronic disease screening and treatment	Personalize risk/Precaution-adoption process model	Present messages as individual and undeniable in a culturally competent and health literate way, and compare them with absolute and normative standards that can be understood by refugee women	MOPH representatives and UNHCR departments receive personal risk feedback on the breast cancer status of refugee women from physicians which will help them realize the extent to which this policy is needed to decrease the burden of the disease among this disadvantaged population
	Arguments/Communication-persuasion matrix	Message new to receiver	Hearing the impact of screening on decreasing the overall burden of breast cancer among refugee women can influence MOPH representatives to sustain the integration of the free mammography section of the Annual Breast Cancer Campaign as part of the policy and developed intervention

### IM Step 4: Program Materials and Educational Components

Step 4 focuses on how the program materials will be created, organized, pretested, and produced based on the matrix of change objectives, the methods, and practical applications previously identified. Taking into account Shioiri-Clark's (43) findings about preferences of refugee populations in receiving educational material, the video series, brochures, bus schedule handouts, appointment calendars, and follow-up material will be developed after involving a multidisciplinary team of experts to pitch-in the generation of drafts. The educational instruments will also integrate the resource-stratified guideline recommendations which stress the need to provide assessments of levels of evidence and thorough details which in turn reflect upon the rationale for the guideline options listed. All materials are culturally relevant and health literate to accommodate for the religious beliefs and educational levels of the refugee population (60, 61).

#### Description of Program Components

The “My Right, My Fight” video series are tailored components that accommodate for health literacy and cultural competence by providing the target audience (refugee women and their families) with basic health information using the Arabic language to disseminate figures, facts, and imagery. The target audience is Syrian and Iraqi refugee women (aged 25 and above) located in the Beirut District camps (main city and suburbs). However, since family support plays a crucial part in the cultural norms and traditions of this community, the videos will be disseminated to the two most commonly used phone applications (Whats app groups and Facebook pages) by refugee women and their husbands (60, 61). The main aim behind these videos is to introduce the target audience to the overall intervention and the severity of the addressed health problem. Three videos will be broadcast across the different social media outlets. A detailed description of each of the video's target audience, sample change objectives, methods (messages and images), and featured people is included in Table 4. Monthly videos of participants will be featured with their families, and continuous awareness public service announcements (PSAs) will be uploaded to answer concerns about screening and chemotherapy (eg., fear of pain, cost, detecting the unknown). These videos will also be shared on morning TV shows to further encourage women to join the fight and claim their right to lead a healthy lifestyle. The methods applied for the videos' change objectives incorporate cues to action and reinforcement through affirmation of covered fees by the credible people included in the video, modeling by religious leaders to appease concerns regarding disrespecting religious beliefs, modeling regarding transportation issues by involving the Minister of Transport who will explain the bus schedule and bus stops that will be set up later on through a collaboration between refugee women and healthcare providers, and elaboration to increase knowledge and correct misconceptions about screening. The producers for this program material consist of a team of graphic designers, health educators, oncologists, translators, interpreters, religious leaders, and community health workers. The involvement of an eclectic team in the design and implementation process will ensure a successful delivery of the tailored health messages. Contracts and budgets will be set between graphic designers and the main funding agencies. Public health professionals, community health workers, and physicians will follow up on the progress of the video production processes to ensure that the initial ideas, sketches, and other proposed visual suggestions are compatible with the culture and values of the target audience on one hand and are effectively transmitting the health messages on the other hand.

**Table 4:** Overview of videos disseminated to the target population.

**Videos**	**Target audience**	**Sample change objectives**	**Methods: messages and images**	**Featured people**
Video 1	Syrian and Iraqi refugee women aged 30 and above located in the Beirut District camps (main city and suburbs)	Adoption of two effective measures to reduce impact of metastatic breast cancer: Screening via mammography Chemotherapy Adherence	Simple figures; facts; statistics emphasizing the burden of the disease at the physical, social, economic, and mental levels A basic guide to explain the different steps of the program Show social support from the different sectors involved in the intervention	Physicians, Community health workers, UNHCR representatives, the Minister of Public Health, public health workers, the Minister of Transport, and Hospital representatives
Video 2	Refugee women and their family members (husbands, elderly)	Increase social support from the elderly and husbands throughout the entire intervention by focusing on the unique role every woman plays in her husband's and children's lives	Appeasing the concerns of refugee women and their families regarding fatalism and seeking medical and preventive measures against God's will	Religious leader (Sheik)
Video 3	Refugee women who did not yet participate in MRMF	Increased perceived susceptibility to breast cancer after watching an educational video about the proper way of carrying out self-examination Decreased perceived barriers toward participation in intervention after listening to testimonials from women of the same culture who survived breast cancer due to screening and proper chemotherapy adherence which can significantly influence the perceived susceptibility beliefs of the refugee women	Success stories from refugee women survivors living within the Middle East region Testimonies from their husbands to motivate refugee women to participate in this intervention Refugee women are asked to share this video with their female friends and relatives All refugees are asked to join the “My Right, My Fight” Facebook group to keep themselves updated with the progress of the project	Refugee women survivors and husbands

In addition to the described video series, billboards that promote the theme, “My Right, My Fight,” will be designed and will target refugee women and their husbands to grab their attention and increase their knowledge, perceived susceptibility, and perceived severity of the health problem, in addition to empowering them to take action by increasing their self-efficacy to engage in screening and treatment measures. The planning group will also design a brochure to be distributed to the refugee women during the educational sessions. The brochure will include an infographic portraying images and messages with information about breast cancer, breast cancer self-examination, and clinical breast examination, steps to follow to reach bus stops, a list of participating hospitals and PHCs (directions and a phone number for reference will be included), fillable appointment calendars to avoid recall bias, and guidelines that should be adhered to for proper follow-up completion. The brochure will also be disseminated on Whats App and Facebook to ensure maximum reach.

Interviews, nominal group technique sessions, and trainings will be held for physicians, public health professionals, community health workers, translators, and interpreters to ensure a detailed understanding of the intervention objectives and to assure the integration of cultural competence and health literacy factors when coming in contact with the target population. These techniques aim to target the self-efficacy of physicians to improve their communication skills with refugee women after undergoing health literacy and cultural competency trainings with expert anthropologists and professional interpreters; their knowledge about the seriousness of breast cancer rates among the disadvantaged population through nominal group techniques in the presence of multiple facilitators who are knowledgeable about the field of refugee health; in addition to addressing their perceived barriers and perceived severity through individual and group interviews to overcome any challenges that might hinder the success of the program. Program planners and the team of producers (graphic designers, video editors, health educators) will organize weekly meetings to certify that all intervention steps are progressing positively and to make any alterations based on the feedback received from focus groups or from the health workers during the preliminary phases of the intervention.

#### Cultural Issues

One of the most essential factors to consider throughout all the steps of intervention mapping is cultural relevance. Cultural issues that need to be addressed specifically when designing the proposed program components pertain to two cultural dimensions: deep structure and surface structure. Deep structure consists of culture factors that influence the behaviors of the target population such as family relationships, religion and ethnic identity, level of acculturation, individuality vs. collectivity, and medical perceptions. In this case, deep structure includes religious and cultural beliefs of Syrian and Iraqi refugees since most of them come from a conservative Muslim background. Seeking screening and treatment measures might be regarded as disrespecting “God's will” or fatalism. Another deep structure factor is family relationships since women tend to seek their husband's approval in matters affecting their personal lives such as medical decisions (15). The level of acculturation and their ethnic identity is also an important factor since many Syrian refugees feel neglected by the Lebanese government and population and are discriminated against when seeking aid in any form (1, 3). For surface structure, this involves the no less important but more superficial cultural aspects of a community such as language, mode of communication, and inclusion of familiar people (21). The intervention will be delivered using the Arabic language; however, translators and interpreters need to make sure that the dialect of the Syrian and Iraqi refugees is taken into consideration to avoid any misunderstanding when delivering the health messages.

#### Increasing Cultural Relevance

To increase cultural relevance for the deep structure factors, a religious leader will be included in one of the videos (Sheik) to explain the importance of seeking preventive and medical care and to appease concerns regarding going against God's will if the refugee women agreed to participate in the intervention. Several representatives from the different involved organizations and institutions are featured in the first video to increase credibility and ensure social support from both the private and public sector in improving access to the needed care for the refugees. The Minister of Transport, the Minister of Public Health, the Head of UNHCR Public Health Division, a religious leader, refugee women breast cancer survivors and their supportive husbands, and a team of public health professionals, community health workers, and physicians will be featured in the video series to emphasize the importance of this intervention in alleviating the burden of the disease. The inclusion of all sectors and actors will encourage refugee women and their husbands to be part of the intervention as they feel wanted rather than neglected by their host community. Both translators and interpreters will be part of the video development to guarantee an effective delivery of health messages in Arabic and avoid any unintentional offensive wording that could affect the credibility of the entire intervention. Informed consent will be taken from all involved parties prior to the development of this interactive media tool.

#### Methods for Pre-testing and Pilot-Testing of Individual and Environmental Prototype Components

##### Concept testing

Concept testing consists of testing key phrases and visuals that emphasize the main ideas of the designed project. Since this intervention plan has been developed primarily based on available literature, it will be essential to conduct concept testing during the development phases of the intervention. To test the images and messages proposed for the videos, billboards, and brochures, focus groups (6–10 members each) will be formulated in camp settings to assess how the target population understands the tailored messages. This feedback will inform any recommended revisions. The focus groups will be formed with the help of public health professionals working with UNHCR since they are deemed credible by the refugee population in camps and their leaders, also known as the Shawishes, and thus will enhance participation in the progress of intervention preparation and implementation by helping in the identification of women who are suffering from breast cancer or who are related to someone diagnosed with the disease. Credibility of organizations from the refugees' perspective is associated with the provision of basic assistance and humanitarian aid. Participation is on a voluntary basis, and women will be informed about the purpose of the discussion and the aim of the intervention in improving their overall health. Additionally, two different versions of the brochure will be tested to see whether refugee women were more comfortable in learning through visuals rather than words and vice-versa. Husbands will be included in separate focus groups to see whether videos and billboards were effective in increasing household-based social support.

To concept test training manuals, physicians, public health professionals, and community health workers will be invited to review the training materials and provide feedback on their effectiveness and acceptability. These trained individuals are employees within the different institutions who act as stakeholders in the development and implementation of the intervention.

##### Readability testing

This pretesting method aims to determine the educational and literacy levels of the target population to design the program materials accordingly. For the sake of this intervention, we will be tailoring the International Adult Literacy Survey to Syrian and Iraqi refugees in Lebanon. This tool is made up of 10 items that were originally developed to determine the literacy level of adult immigrants coming into the U.S (National Center for Education Statistics, n.d). The readability of the informational brochures, bus schedules, appointment calendars, and follow-up material provided by the physicians will all be tailored to the educational level of the majority of Iraqi and Syrian refugees in the targeted camps.

##### Message execution

This form of testing seeks to assure that the program materials and messages are relevant, culturally acceptable, and comprehensible by the target population. Brochures, billboards, and video assessments will be shared with groups of refugee women and their husbands by organizing focus group sessions and interviews with community health workers and translators. However, prior to the distribution of the intervention items to refugees, the program planners, interpreters, translators, and the team of producers will make sure that the written messages incorporate a cultural perspective to ensure effective communication through the selected delivery vehicles and will include the concept of decentering in translation to justify the choice of words selected in the foreign language to convene the desired health message. The presentation of medical terminology in the program materials will be simplified to promote processing of information and allow for improved understanding of the benefits of screening and the diagnosis and treatment processes of breast cancer. The design of the print material will be reviewed to sustain compatibility between the intervention objectives and the delivery vehicles and to avoid any unnecessary diversions. As for the videos, the scripts and storyboards will be edited before the pre-testing and pilot testing phase to ensure that all elements of the design document are met and that both the planning team and production team are on the same page. Table 5 includes example questions that will be asked during the pretesting/pilot testing phase.

**Table 5:** Example questions for pretesting and pilot testing phases.

**Target population**	**Pretesting/pilot testing questions**
Refugee women	Please tell me in your own words, what are the key messages in the brochures and flyers about breast cancer? How likely are you to participate in the intervention? Are there any words or images in the materials that are difficult to understand? Should we include more pictures? What are the best ways to get this information to refugee women? Prompt for social media, e.g., Whats App, Facebook Tell me about the women featured in the video. How similar are they to you or to other women that you know? How does their story affect you? Is there anything in the program materials that makes you uncomfortable or upset? Please explain why. What, if anything, could we do to make these materials more appealing and impactful?
Refugee Husbands	After watching the video and reading the print materials, how serious a problem do you think breast cancer is for women? How likely is it that your wife would participate in the intervention? Is there anything in the program materials that makes you uncomfortable or upset? Please explain why. What, if anything, could we do to make these materials more appealing and impactful? How likely is it that you will support and help your wife during the different intervention steps, including the time she has to go to the hospital to access the needed care?
Health providers/physicians/translators & interpreters/ public health workers	Please share your thoughts about the intervention guide. How easy is it to explain the information to the refugee women? What parts of the material need to be altered? How feasible is it for you to adjust your schedule and include a slot for refugee women based on their time preference and the bus schedule set by the Ministry of Transport? How feasible is it for you to work collaboratively with interpreters and translators without causing confusion, mistrust, and loss of credibility? Do you think the video sends an appropriate message for refugee women? Why or why not? What, if anything, should we change to make the video more impactful?
UNHCR Representatives	What are the challenges you faced during the preliminary phase of the intervention in terms of funding, gaining the attention of refugees, and recruiting the needed healthcare personnel? Do you think that any of these challenges pertain to the way messages are being delivered? Is there anything that needs to be changed? Does the video highlight all the sectors and sponsors involved in the intervention? Are any of the video messages in need of modification? Does the information regarding the goals and objectives of the intervention appeal to you and to the overall organizational culture? Do you think that the billboards and flyers are sending the right message and will gain the attention of a wide audience? Do you think that the program materials will increase acceptability from the host community concerning the implementation of the intervention for Iraqi and Syrian refugee women in Lebanon?
MOPH Representatives	What are the challenges you faced during the preliminary phases of drafting the policy in terms of getting the support of the parliament, the ministers, and the Lebanese community at large? Do you think that any of these challenges pertain to the way messages are being delivered about the policy? Is there anything that needs to be changed? Is the information regarding the goals and objectives of the intervention compatible with what the policy aims to achieve over the long-term? How well do you think that the program materials will increase acceptability from the host community regarding the execution of the policy at a national level?

##### Impact

A questionnaire evaluating each of the determinants targeted by the different intervention components will be distributed to refugee women during focus group sessions and preliminary phases of the intervention to assess the impact of program change methods. The collected data will give us an idea if any corrective actions need to be taken for the selected change methods.

##### Adoption/implementation characteristics

The purpose of this final step is to predict any potential problems with implementation. To determine the perceptions of implementers toward complexity, trialability, observability, and relative advantage of materials, separate focus groups will be conducted for the different sectors involved in the intervention including the intrapersonal (refugee women), interpersonal (physicians), organizational (UNHCR), and societal levels (MOPH) along with the other members of the planning group. To evaluate trial implementation, the research team will collect qualitative data through observation and ethnographic techniques regarding how well the refugee women are responding to the print and media material, how the communication process is going between physicians and target populations while having interpreters and translators as a third party mediator, how well the coordination of events and the funding of services is happening at the organizational level, how smoothly the policy execution process is going, and how motivated refugee women are to participate in all steps of the intervention after adequately understanding the impact of the problem through culturally relevant and health literate communication processes. Information tracking the evolution of the policy process and the passing of the draft in parliament will also be updated continuously to detect and deal with unexpected and expected challenges at an early stage. The data will then be shared with all members of the planning group to carry out the necessary changes.

## Discussion

This article delineates an intervention plan to increase breast cancer screening and chemotherapy adherence among Syrian and Iraqi refugee women residing in refugee camps in Beirut. It also provides future public health workers and research experts with an intervention plan for a concerning health issue in Lebanon that is disproportionately affecting disadvantaged populations in the country, specifically refugees. High incidence and prevalence rates of metastatic breast cancer among Iraqi and Syrian refugee women should be urgently addressed in camp settings since the limited funds allocated for the management of chronic diseases among asylum seekers in Lebanon renders the diagnosis of breast cancer at an early stage currently impossible (3, 11, 17). Therefore, implementing a health promotion intervention using the intervention mapping approach can integrate the multiple disciplines of public health to increase the adoption of the targeted health behaviors (mammography, chemotherapy adherence, proper follow-up measures) and subsequently reduce the overall financial, social, and health burden of the disease which not only inadvertently impacts refugee populations but also the host community at large (1, 3, 6, 11). Since the intervention has not yet been implemented, IM Steps 5 (Designing an Implementation Plan) and 6 (Creating an Evaluation Plan) will be described seperately after completion of the program. The rationale behind using IM is the integration of both the theoretical aspects of health promotion along with the evidence and new data from the literature to find sustainable solutions that address the personal and environmental determinants using a socioecological perspective. IM was also helpful in selecting the most effective theory-based change methods and appropriate practical applications, in addition to clarifying multiple factors that should be taken into consideration when designing and creating educational program components.

The developed “My Right, My Fight” (MRMF) program targeted one primary behavioral and three environmental outcomes which were deemed most effective in addressing high rates of breast cancer. Both mammography and self-examination of nodules contribute to the early detection of cases and to increasing positive response rates to treatment (46–48). The interpersonal and organizational environmental outcomes will play a crucial role in ensuring the overall success of the intervention and in attainment of the desired health behaviors (63). Having UNHCR support diagnostic and treatment measures through an increase in the allocation of funds for refugee chronic disease management and creation of supportive and trustworthy patient-physician relationships which take into account the cultural norms of the refugee population will be an essential factor in ensuring the sustainability of the program and the targeted health outcomes based on previous research studies and intervention projects (12, 19, 49, 50). Moreover, the creation and execution of a comprehensive policy at the societal level which protects the rights of refugees in accessing chronic disease screening and treatment services and encompasses the options as depicted by the internationally recognized resource-stratified guidelines is also a major key factor in determining the long-term success of the intervention (3, 20, 51, 63).

This intervention was based on a culturally competent and health literate approach to emphasize respect and acknowledgment of the refugee women's norms and perceptions. The different steps of IM integrated within “My Right, My Fight” will take into account cultural sensitivity to increase the credibility of the program and to foster high retention rates along the entire process (1, 19, 21).

## Limitations

Due to the limited quantitative studies available about this particular topic, the following intervention has been based mostly on the needs assessment carried out by looking at qualitative research studies targeting refugee populations in Lebanon and the surrounding Middle Eastern region. Despite it being mainly theoretical, we believe that the application of the IM approach to our developed intervention will greatly contribute to the effectiveness of “My Right, My Fight” (MRMF) since this plan acts as a guide for a team of multidisciplinary health experts working with refugee populations in third world countries (52, 64). The incorporation of the resource-stratified guidelines adds credibility and elevates the chances of success and sustainability as emphasized in the literature (3, 20, 51). However, due to the political instability reigning over the Middle East region including Lebanon, many challenges (expected and unexpected) can take their toll on the advancement and fulfillment of the program, especially in terms of allocation of funds on a long-term basis (65). Additional external sponsors will be needed, and a collaboration of the different local and international agencies will impart the support needed for the continued adoption and implementation of the program.

## Data Availability Statement

The raw data supporting the conclusions of this article will be made available by the authors, without undue reservation, to any qualified researcher.

## Author Contributions

LS and CM: development of the program plan and manuscript development and primary writers. JF: critical review of manuscript.

### Conflict of Interest

The authors declare that the research was conducted in the absence of any commercial or financial relationships that could be construed as a potential conflict of interest.
